# The Effect on Muscle Activity of Reaching Beyond Arm's Length on a Mobile Seat: A Pilot Study for Trunk Control Training for People After Stroke

**DOI:** 10.1016/j.arrct.2023.100289

**Published:** 2023-08-07

**Authors:** Bettina Sommer, Michelle Haas, Samuel Karrer, Matthias Jörger, Eveline Graf, Martin Huber, Daniel Baumgartner, Jens Bansi, Jan Kool, Christoph Bauer

**Affiliations:** a School of Health Sciences, Zurich University of Applied Sciences, Winterthur, Switzerland; b School of Engineering, Zurich University of Applied Sciences, Winterthur, Switzerland; c Physiotherapy Department, Valens Rehabilitation Centre, Valens, Switzerland; d OST, University of Applied Sciences of Eastern Switzerland, St. Gallen, Switzerland

**Keywords:** Biomechanical phenomena, Electromyography, Exercise therapy, Rehabilitation, Stroke rehabilitation

## Abstract

**Objective:**

This pilot study compared muscle activity during lateral reaching tasks between mobile and stable sitting using a novel therapy chair in people after stroke and healthy controls.

**Design:**

Observational pilot study.

**Setting:**

This study was conducted in a rehabilitation center for people after stroke and at the university's movement laboratory for healthy participants.

**Participants:**

A total of eleven people after stroke and fifteen healthy people (N=26) took part.

**Interventions:**

Lateral reaching exercises to the ipsilateral and contralateral sides were performed on a mobile and a stable seat.

**Main Outcome Measure:**

Muscular activity of the multifidus, erector spinae and external oblique was measured bilaterally. A within-subject linear mixed model was applied to analyze the effects of seat condition, task, muscle side, and group.

**Results:**

A seat condition effect was found for the multifidus and external oblique that was dependent on the muscle side and task. During ipsilateral reaching, the activity of the multifidi decreased for people after stroke on the mobile seat, while increasing for healthy participants. The erector spinae showed no condition effect. Decreased activity of the external oblique was found for both groups on the mobile seat.

**Conclusions:**

Mobile sitting influences muscular activity. However, these preliminary results should be further investigated in order to generate recommendations for rehabilitation.

The ability to control posture while sitting or standing is essential in executing activities of daily living, such as dressing, reaching for an object, or walking. It is a fundamental prerequisite to living an independent everyday life.^1^^,^^2^ Trunk control plays a central role in controlling posture because the trunk connects the upper extremity with the lower extremity. A variety of conditions, such as aging^3^ or neurologic disease (eg, stroke),^4^ can cause trunk control impairment and it is 1 of the key factors addressed during stroke rehabilitation.^5^ It has already been shown that trunk exercises can improve trunk control and lead to better balance and mobility in people after stroke.^1^^,^^6^ Trunk control is highly associated with trunk muscles, which allow the trunk to remain in an upright, stable position during movements of the upper body.^7^^,^^8^ Therefore, trunk muscular activity can be measured as an indicator of trunk control.

After a stroke, patients can sit before being able to stand. Therefore, an early application of trunk control training in a seated position is beneficial. Such seated training during neurorehabilitation often includes reaching tasks beyond arm's length. The effectiveness of this training in improving trunk control has been demonstrated in several studies.^1^^,^^9^, ^10^, ^11^ Lateral reaching beyond arm's length appears to require greater trunk control than frontal reaching. Compared to frontal reaching, in lateral reaching the center of mass shifts away from the support base earlier and there is no support from the feet.^12^ Lateral and diagonal reaching beyond arm's length are therefore frequently used tasks to improve trunk control in stroke rehabilitation.^13^^,^^14^

Reaching tasks in a seated position can be performed either on a mobile or stable surface. Studies have shown that, compared to a stable surface, the performance of trunk exercises on a mobile surface leads to a greater improvement in trunk control.^11^^,^^15^ Training on a mobile surface (eg, a physiotherapy ball)^16^ is beneficial, however, currently available tools are impractical during early rehabilitation. These tools show a higher risk and fear of falling, combined with a time-consuming and costly supervision effort. To the best of our knowledge, there is no training tool currently used during early neurorehabilitation that adequately ensures patient safety during seated, mobile trunk control training. A mobile therapy chair (T-Chair) was previously developed to meet this need and enable safe and repetitive trunk training.^17^ The ability to configure the seat surface of the T-Chair as either stable or mobile allows adaptation of the intensity to the user's need. User movement actively controls the mobile seat surface, while patient safety is provided through the availability of armrests, back support, and a seat belt. The mobile T-Chair facilitates the safe performance of a variety of trunk control improvement tasks, such as dynamic reaching tasks beyond arm's length. The T-Chair has the potential to be an effective tool to train trunk control in people early after stroke.^17^

People after stroke are known to have impaired trunk musculature.^18^^,^^19^ In healthy participants, the trunk muscles have been shown to be increasingly active with decreasing seat stability.^20^ Therefore, a stabilization training through mobile sitting could enhance muscular activity in people after stroke.

The aim of this pilot study was to compare maximal trunk muscle activity during lateral reaching tasks between mobile and stable sitting using the T-Chair in people after stroke and healthy participants.

## Methods

This pilot study was conducted as a cross-sectional, observational study. As it is the first study to investigate muscular activity on this mobile seat, no prior sample size calculation was possible.

### Participants

General inclusion criteria for all participants were: age of at least 18 years; body mass index between 18 to 28 kg/m^2^; no pregnancy.

People after stroke were recruited from the inpatient rehabilitation clinic Valens (Switzerland) and had to be without any other acute or chronic diseases (eg, back pain), able to understand instructions, and perform at least 2 hours of rehabilitation training per day. A further inclusion criterion for people after stroke was a Trunk Impairment Scale score of between 2 and 19 points representing at least minimal impairment but independent sitting. Healthy participants were recruited from staff at the university and by word-of-mouth advertising. They had to be free of any musculoskeletal, neurologic, or cardiopulmonary diseases and have no amputations or scoliosis. The ethics committee of Zurich juristically verified the pilot study (Req-2020-00569) and all participants signed an informed consent form.

### Investigated Product

**Fig 1:**
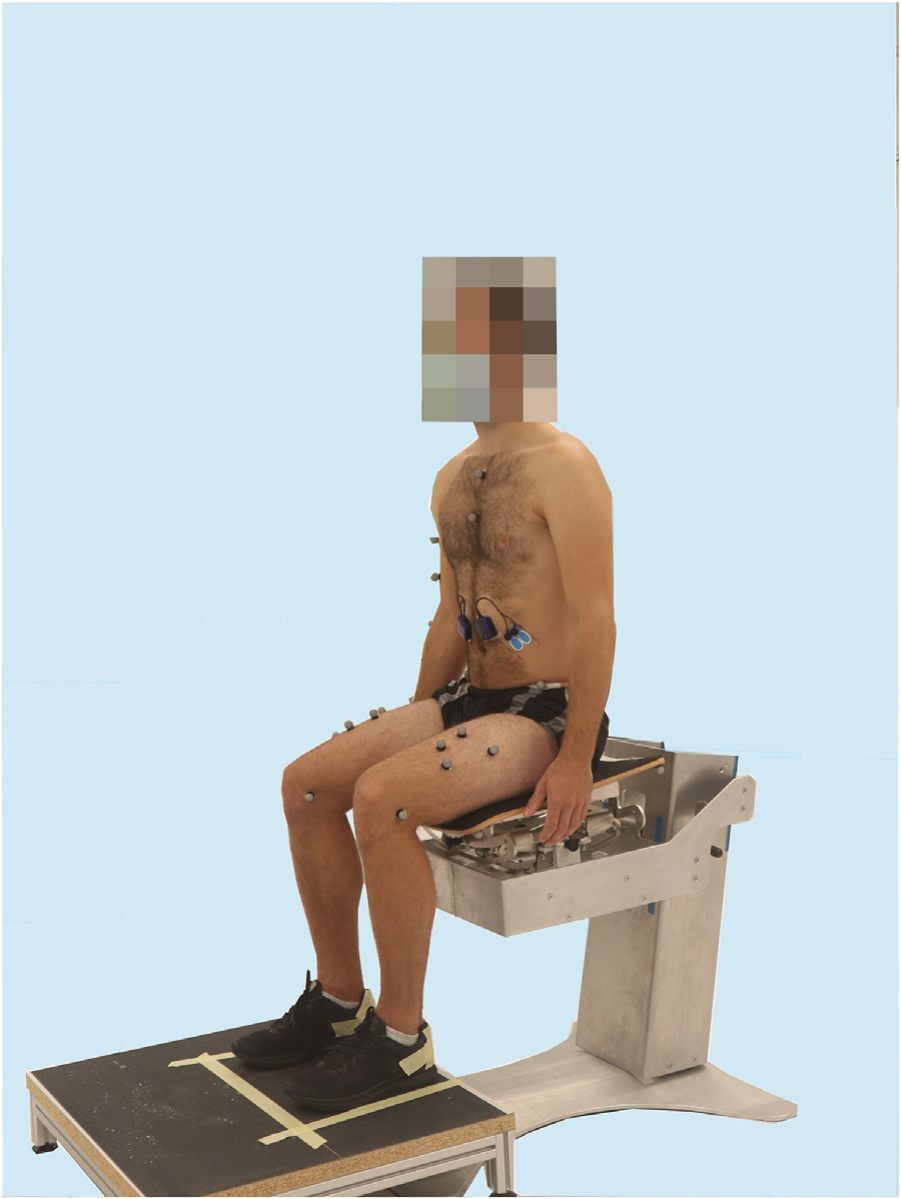
Participant sitting in the starting position on the mobile / stable seat.

The exercises were performed on a seat with a movable surface (fig 1), which has been described in detail in earlier studies.^17^^,^^21^ The seat was developed specifically for the purpose of trunk control rehabilitation after stroke. The seat surface can be moved in medio-lateral and antero-posterior directions, or in a combination thereof. The design allows the lower spine and pelvis to move while the upper spine remains stable. The virtual rotation axis is located at the spine.^22^, ^23^, ^24^ Safety requirements were implemented for its use in therapy according to current technical standards.^25^, ^26^, ^27^ The seat used for the healthy participants in this pilot study slightly deviates to the 1 used for people after stroke. In the laboratory setting for healthy participants, a prototype was used with no backrest to allow visibility of the spine.

The movable seat surface can be locked to achieve a stable surface. The locked position is referred to as ‘stable sitting’ and the unlocked, moveable position as ‘mobile sitting’. A simple mechanism to change between stable and mobile sitting allows either position to be used for training. Both stable sitting and mobile sitting were examined in this study. When unlocked, a person can actively control the movement of the seat surface.

### Data collection

Data collection of the people after stroke took place at the Valens clinic and that of healthy participants at the movement laboratory of the Zurich University of Applied Sciences. Muscle activity was measured using surface electromyography (EMG) with a wireless transmitter at 1200 Hz (Myon AG, Baar, Switzerland^a^). The skin at the determined electrode location was first shaved and disinfecting. Then, participants were equipped with electrodes^b^ for the muscles multifidus (MF), erector spinae (ES), and external oblique (EO). Electrodes were placed on both sides, according to SENIAM^28^ for MF and ES and according to Ng et al^29^ for EO. Electrodes were applied by experienced staff (3 physiotherapists, 1 human movement scientist). Using guidelines, intra-individual differences were minimized.

**Fig 2:**
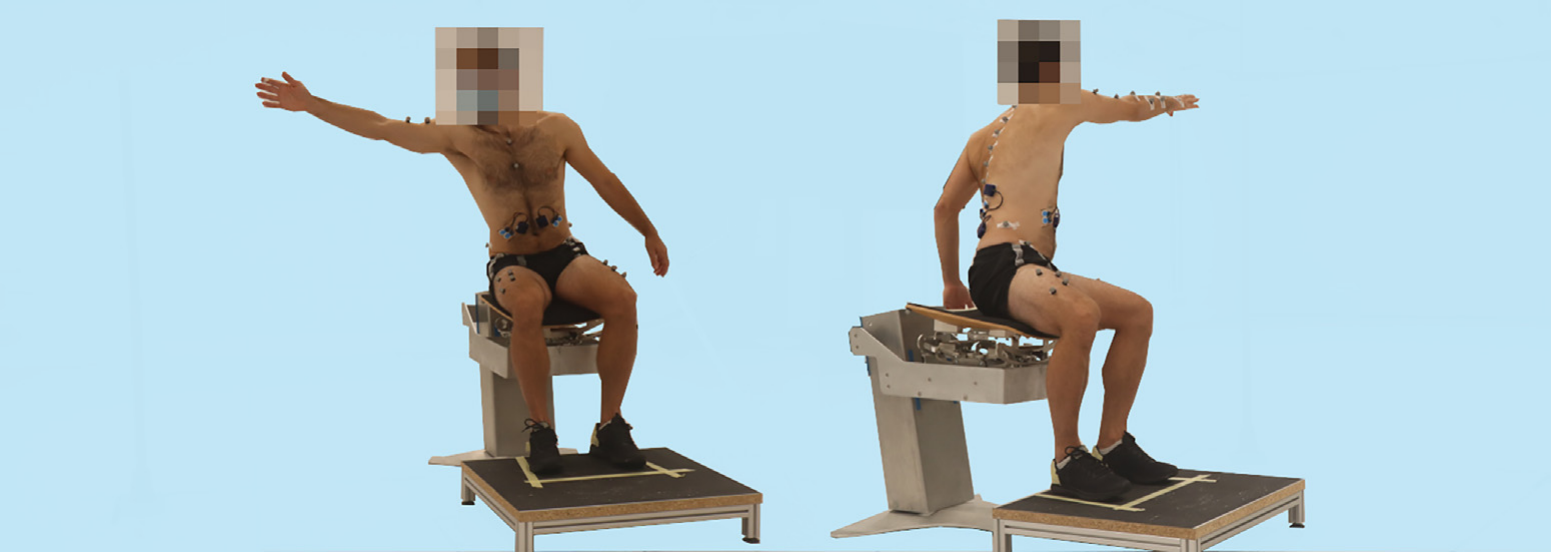
Reaching to the ipsilateral side (left picture) and reaching to the contralateral side (right picture) on the mobile seat.

At the beginning of each session, a static trial in the stable seated position was captured for a few seconds. Participants were asked to sit upright with rectangular hip and knee angles, feet hip-width apart and arms hanging loosely beside the body. Participants were then asked to reach beyond arm's length in the ipsilateral sidewards direction and the contralateral diagonal direction (fig 2). The target direction was marked with a pilone, which was placed in the direction where participants should reach. People after stroke performed the reaching with their unaffected arm, while healthy participants used their dominant arm (hereafter ‘unaffected’). The feet always remained on the ground. The non-dominant or affected arm (hereafter ‘affected’) rested on the lap. The seat height was adjusted until the knees and hips were at 90° angles and the feet were placed hip-width apart on the pedestal. Each task was performed 5 times on the mobile and the stable sitting surfaces, respectively.

### Data analysis

EMG data were captured using Vicon Nexus^c^ in the laboratory and proEMG^a^ (Myon AG, Baar, Switzerland) in the clinic. For processing, raw signals were exported and further processed in Matlab^d^. EMG signals were filtered with a second order Butterworth filter, with a cut-off frequency of 30 Hz for high-pass and 500 Hz for low-pass filtering. The root mean square window was 120 frames. All EMG data were normalized to the maximal activity in static sitting. EMG data of each trial were visually checked by 2 persons for artifacts or missing signal. Maximal EMG activity was defined as the maximal value during a trial. For further data analysis of the dynamic trials, the mean of the 5 trials maxima for each muscle was taken.

Statistical analysis was performed in R Software^e^ with the packages lme4,^30^ lmerTest,^31^ psych,^32^ emmeans,^33^ and ggplot2.^34^ A 3-way within-subject linear mixed model was applied, with 1 between-subject covariate group (people after stroke/healthy), as well as the within-subject covariates of muscle side (unaffected/affected), seat condition (mobile/stable) and task (ipsilateral/contralateral). The model was fitted to the EMG data for each muscle separately. The outcome Y_i_ is the normalized maximal EMG activity of each muscle, $\beta_{0}$ the intercept, $\beta_{k}$ the effect of the covariate k, and $\varepsilon_{i}$ the independent and normal distributed error. A significance level of *P*<.05 was applied. All parameters were logarithmically transformed to reach normal distribution of the residuals. For better interpretation, the outcome was transformed back and is therefore reported non-logarithmically in the results.

$$Y_{i}=\beta_{0}+\beta_{1}Group_{i}+\beta_{2}Side_{j}+\beta_{3}Condition_{j}+\;\beta_{4}Task_{j}+\beta_{5}Group_{i}xSide_{j}\;+\beta_{6}Group_{i}xCondition_{j}+\beta_{7}Group_{j}xTask_{j}+\;\beta_{8}Side_{j}xCondition_{j}+\beta_{9}Side_{j}xTask_{j}+\beta_{10}Task_{j}xCondition_{j}+\;\beta_{11}Group_{i}xSide_{j}xCondition_{j}+\;\beta_{12}Group_{i}xSide_{j}xTask_{j}+\beta_{13}Group_{i}xTask_{j}xCondition_{j}+\;\beta_{14}Task_{j}xSide_{j}xCondition_{j}+\beta_{15}Group_{i}xTask_{j}xSide_{j}xCondition_{j}\;+\;\varepsilon_{i}$$

The final model was determined using a stepwise model selection procedure with backwards optimization where non-significant covariates were excluded. The aim of this procedure was to choose a parsimonious model to prevent overfitting of the data. This procedure ensured optimization of the model for the prediction of future data. The fitted model was used to predict EMG activity. The final models used are presented in table 1.

**Table 1:** Final statistical model for each muscle

Muscle	Final model
Multifidus	without 4-way interaction $\beta_{15}Group_{i}xTask_{j}xSide_{j}xCondition_{j}\;$and without $\beta_{3}Condition_{j}$
Erector spinae	without 4-way interaction $\beta_{15}Group_{i}xTask_{j}xSide_{j}xCondition_{j}$, without 3-way interactions $\beta_{11}Group_{i}xSide_{j}xCondition_{j},\;\beta_{12}Group_{i}xSide_{j}xTask_{j},\;\beta_{13}Group_{i}xTask_{j}xCondition_{j},\;\beta_{14}Task_{j}xSide_{j}xCondition_{j}$and without $\beta_{3}Condition_{j}$
External oblique	without 4-way interaction $\beta_{15}Group_{i}xTask_{j}xSide_{j}xCondition_{j}$

The results correspond to the predicted mean normalized maximal EMG activity, according to the fitted model. In addition, for each covariate, contrast ratios were calculated, for example, the condition ratio of the EMG activity of mobile compared to stable sitting.

$$\text{condition}\;\text{ratio}=\;\frac{Y_{i,\;\text{mobile}}}{Y_{i,\;\text{stable}}}$$

## Results

A total of 26 participants (11 people after stroke, 15 healthy) were included for analysis. Of the 11 people after stroke (5 females and 6 males), 6 had a dominant / less affected right side. Of the 15 healthy participants (5 females and 10 males), 13 had a dominant (unaffected) right arm. Further descriptive values of the study population are listed in table 2. Missing data arose from a wrongly placed electrode for EO (1 healthy person), from being too exhausted to perform contralateral reaching (1 person after stroke), and from missing data entry on a participant's Trunk Impairment Scale score (1 person after stroke). Missing data were replaced by NaN (Not a Number).

**Table 2:** Characteristics of the study population

Characteristics	People after stroke		Healthy	
	n	Mean ± SD	n	Mean ± SD
Body mass index (kg/m^2^)	11	22.3 (3.8)	15	23.0 (1.6)
Age (y)	11	70.2 (14.7)	15	29.9 (5.5)
Trunk Impairment Scale (points) • Static sitting • Dynamic sitting • Coordination	10	13.7 (2.7) 5.6 (0.5) 5.7 (1.7) 2.4 (1.4)		

The data presented in this section correspond to the predicted EMG activities - see table 3 and appendix 1. The real EMG activities, respective descriptive values, can be found in appendix 2.

**Table 3:** Predicted means of normalized maximal EMG activity of each muscle [% static]; unaffected side corresponds to the dominant side in healthy participants, affected side to the non-dominant side

			Multifidi				Erector spinae				External oblique			
			People after stroke		Healthy		People after stroke		Healthy		People after stroke		Healthy	
Task	Condition	Side	Mean	CI	Mean	CI	Mean	CI	Mean	CI	Mean	CI	Mean	CI
Ipsilateral reaching	Mobile	Unaffected	120.3	82.3-175.8	160.4	115.9-222.2	121.4	82.9-177.6	186.1	133.9-258.7	163.8	114.6-234.1	312.7	230.0-425.2
		Affected	162.7	111.3-237.7	368.9	266.3-510.8	301.2	205.9-440.8	411.2	295.9-571.6	121.9	84.1-176.6	221.2	162.7-300.8
	Stable	Unaffected	162.5	111.2-237.4	138.0	99.2-191.9	126.3	86.3-184.8	202.7	145.8-281.7	119.0	83.3-170.2	236.4	173.9-321.5
		Affected	243.8	166.9-356.3	332.6	240.2-460.6	306.1	209.2-447.9	437.3	314.6-607.9	179.9	124.1-260.7	347.9	255.8-473.1
Contralateral reaching	Mobile	Unaffected	256.0	173.0-378.9	539.6	389.7-747.4	209.5	142.3-308.4	464.0	333.8-644.9	162.5	112.3-235.2	378.2	278.1-514.2
		Affected	158.3	107.0-234.4	455.3	328.8-630.6	294.8	200.2-434.0	581.2	418.1-807.8	164.4	111.9-241.7	188.5	138.5-256.1
	Stable	Unaffected	321.6	215.6-479.7	771.1	556.7-1067.8	222.0	150.8-326.9	514.7	370.3-715.4	191.7	132.5-277.4	396.1	291.3-538.6
		Affected	140.8	95.1-208.5	435.2	314.2-602.7	305.1	207.3-449.2	629.6	452.9-875.1	191.1	130.1-280.9	199.1	146.4-270.8

Abbreviations: CI, confidence interval.

### Multifidus (MF)

People after stroke showed lower MF activity on the mobile than on the stable seat, except for the contralateral task on the affected side (table 4). Conversely, healthy participants exhibited greater MF activity on the mobile than on the stable surface, except for the contralateral task on the unaffected side. As seen from the interaction effects, condition interacts with side and task and with group and task.

**Table 4:** Condition ratios of mobile to stable sitting for each task, group, and side; unaffected side corresponds to the dominant side in healthy participants, affected side to the non-dominant side

		Multifidi				Erector spinae				External oblique			
		People after stroke		Healthy		People after stroke		Healthy		People after stroke		Healthy	
Task	Side	Condition Ratio (mobile/stable)	CI	Condition Ratio (mobile/stable)	CI	Condition Ratio (mobile/stable)	CI	Condition Ratio (mobile/stable)	CI	Condition Ratio (mobile/stable)	CI	Condition Ratio (mobile/stable)	CI
Ipsilateral reaching	Unaffected	0.74	0.57-0.97	1.16	0.91-1.48	0.96	0.77-1.20	0.92	0.75-1.13	1.38	0.97-1.96	1.32	0.97-1.80
	Affected	0.67	0.51-0.87	1.11	0.88-1.40	0.98	0.79-1.23	0.94	0.77-1.16	0.68	0.47-0.98	0.64	0.47-0.87
Contralateral reaching	Unaffected	0.80	0.60-1.06	0.70	0.55-0.89	0.94	0.75-1.19	0.90	0.73-1.11	0.85	0.59-1.23	0.96	0.70-1.30
	Affected	1.12	0.85-1.49	1.05	0.83-1.32	0.97	0.77-1.21	0.92	0.75-1.14	0.86	0.59-1.27	0.95	0.70-1.29

Abbreviations: CI, confidence interval.

For MF, significant 3-way interactions were found for condition:group:task (*P*=.004) and condition:side:task (*P*=.022). Additionally, 2-way interactions existed for side:task (*P*<.001), condition:task (*P*=.002) and condition:side (*P*=.012), as well as main effects for task (*P*<.001), group (*P*=.004) and condition (*P*=.003). All predicted means are shown in table 3.

Regarding the tasks, contralateral reaching showed more MF activity than ipsilateral reaching, except on the people after stroke group's affected side. Contralateral reaching showed more involvement of the unaffected side, while ipsilateral reaching involved the affected side more. People after stroke showed less MF activity than healthy participants, except for ipsilateral reaching on the stable surface.

### Erector spinae (ES)

There was no significant difference between the mobile and stable surfaces for all participants (table 4). Therefore, this condition does not influence ES activity.

Significant effects were found for group:task (*P*=.009), side:task (*P*<.001), as well as task (*P*<.001) and side (*P*<.001). All predicted means are shown in table 3.

Contralateral reaching generated more ES activity than ipsilateral reaching, except for the after stroke affected sides. Generally, the affected side is more active than the unaffected side for both contralateral and ipsilateral reaching tasks. People after stroke show lower activity than healthy participants do for the contralateral and ipsilateral tasks for the affected side. The ipsilateral unaffected side generally has low ES activity, which is slightly increased in people after stroke compared to healthy participants.

### External oblique

The mobile condition predominantly led to reduced EO activity, except for the ipsilateral reach of the unaffected side (table 4).

Significant effects exist for side:group:task (*P*=.011), condition:side:task (*P*=.004), side:group (*P*=.006), group (*P*<.001) and side (*P*<.001). All predicted means can be found in table 3.

Contralateral reaching showed higher EO activity than ipsilateral reaching, except for the affected side of healthy participants. The unaffected side was more active than the affected side for contralateral reaching and ipsilateral reaching on the mobile surface. All people after stroke had lower EO activity than healthy participants.

## Discussion

The main goal of this pilot study was to examine the effect of the mobile sitting condition on lateral reaching tasks. The results indicate that reaching exercises for people after stroke can be performed on both stable and mobile seats. The mobile condition shows an effect on MF and EO activity. Since ipsilateral reaching exhibits lower activity, this exercise should be used earlier in rehabilitation than contralateral reaching, which could be added later. The mobile sitting condition predominantly showed lower activity for people after stroke, and this could benefit rehabilitation in its early stages.

While ES showed no significant condition effect, MF and EO had a condition effect that was dependent on the side and task. MF were shown to be involved in the stabilization of the spine.^35^ However, the effect of a mobile sitting condition remains unclear. While an earlier study found no change in muscular activity between the stable and mobile sitting conditions,^36^ some changes in muscular activity due to seat condition were found in this pilot study. The difference could arise from the different types of mobile condition. While O'Sullivan et al^36^ used an air-filled cushion with the rotation axis on the lumbar spine, this pilot study used a pivotable seating surface with a rotation axis on the lumbo-thoracic junction. Therefore, the design and type of mobile condition applied seems important. On the other hand, the unchanged activity of ES might be due to the sidewards movements, in which ES seems to be less involved. This fits to the findings that stabilization exercises to the side need less ES activity than a bridging exercise.^37^ For EO activity, our findings with less muscular activity on the mobile seat match the findings that the change in muscular activity only occurs under static spine stability, but not under dynamic stability.^38^ This concurs also with the findings of a static desk work leading to increased EO activity with an unstable dual foot support.^39^

For MF in ipsilateral reaching, people after stroke showed less activity on the mobile surface compared to the stable surface, while healthy participants exhibited more activity on the mobile surface. This group difference might arise from the fact that healthy participants move in a larger range and are, therefore, more affected by the seat condition. Additionally, core stability is known to be impaired in people after stroke.^40^^,^^41^ Since training on a mobile seat has been shown to be effective,^15^^,^^40^ it would be interesting to see whether this group difference diminishes after a period of training.

The task difference of ipsilateral and contralateral reaching showed an effect for the back muscles MF and ES (main effect and interaction effects), as well as an interaction effect for EO. Our results showed that ipsilateral reaching needs less muscle activity and is therefore more suitable for use in early rehabilitation than contralateral reaching. The lower activity could be due to a smaller movement range. It has been shown that people after stroke, through keeping the body as stable as possible and moving more with the upper parts of the trunk, display a smaller shift of their center of pressure during trunk movements.^42^ This effect is thought to be enhanced with ipsilateral reaching, where the limit of stability is reached sooner than with contralateral reaching.

The tasks performed in this pilot study were unilateral tasks. Side differences in unilateral tasks when compared to bilateral movements, have been observed in a previous study.^43^ Similarly, in our pilot study side differences of the muscles were observed. This information helps in choosing the task to train a specific body region most effectively. For example, to train the affected side, ipsilateral reaching would be more effective, as it leads to higher muscular activity than contralateral reaching.

### Study Limitations

Nevertheless, some limitations need to be considered. First, because of the small sample size of this pilot study our results are not generalizable. Particularly for people after stroke, where impairment status differs, a larger sample needs to be tested to indicate whether these results are generalizable. Because of the COVID-19 pandemic, the recruitment of participants was restricted, both in terms of sample size and matching between the groups. This resulted in the groups differing substantially in terms of age, which could have an influence on trunk control. Also, measuring electromyographic activity can cause some erroneous measurements. In order to decrease measurement error, standardized procedures and training of the study personnel was performed. The mean across trials also minimizes outliers. Another limitation is the measurement on a single training session, which does not allow insight into the long-term training effects.

## Conclusion

Seat condition was found to influence muscular activity. As a next step, reaching exercises on the mobile surface should be investigated at 2 points in time, to determine the training effects further. Further predictive factors, like the early prediction of functional outcomes after stroke (EPOS), could be considered.^44^ Also, the relation between time since stroke, age and muscle activity could be analyzed with a larger patient group.

## Suppliers

a. proEMG; Myon AG.
b. Blue Sensor; Ambu.
c. Vicon Nexus Version 2.11; Vicon.
d. Matlab Version R2019a; Mathworks.
e. R Version 4.1.0; R Core Team.

## Figures and Tables

**Table:** 

					Multifidus [% static]		Erector spinae [% static]		External oblique [% static]	
Task	Group	Condition	Side	No.	Mean	SD	Mean	SD	Mean	SD
Ipsilateral reaching	Patient	Mobile	Unaffected	11	137.21	74.73	135.38	78.97	180.71	103.94
			Affected	11	212.96	176.34	363.47	225.65	149.76	75.48
		Stable	Unaffected	11	202.42	166.23	151.95	93.62	152.06	92.34
			Affected	11	281.74	156.77	405.17	251.64	177.35	60.27
	Healthy	Mobile	Unaffected	15	188.57	104.33	246.09	194.25	352.09	138.86
			Affected	15	469.31	370.75	496.17	329.18	229.58	92.72
		Stable	Unaffected	15	151.09	44.40	221.99	131.97	282.21	178.80
			Affected	15	416.68	309.89	600.52	552.49	593.64	1019.67
Contralateral reaching	Patient	Mobile	Unaffected	10	383.90	417.05	228.49	113.49	216.51	150.84
			Affected	10	176.88	110.37	336.68	198.45	197.11	132.32
		Stable	Unaffected	10	467.45	444.02	236.94	158.16	215.40	127.70
			Affected	10	179.06	161.08	336.97	219.35	223.40	118.42
	Healthy	Mobile	Unaffected	15	620.18	332.71	520.02	305.33	440.76	287.22
			Affected	15	517.78	274.77	723.94	449.60	211.73	90.07
		Stable	Unaffected	15	909.52	480.70	657.65	382.19	527.50	542.79
			Affected	15	528.22	357.53	730.90	397.24	214.02	108.09

## Appendix 1 Contrast Plots

Black point represents Mean, gray bar the confidence interval; unaffected side equals the dominant side in healthy participants, while affected equals the non-dominant side.

**Multifidi**

**Erector spinae**

**External oblique**

## Appendix 2 Descriptive Values of Each Muscle, Reported as Maximal EMG Activity in % of Static Sitting

Side: unaffected equals the dominant side in healthy participants, while affected equals the non-dominant side

## References

[1] Dean CM, Shepherd RB (1997). Task-related training improves performance of seated reaching tasks after stroke. Stroke.

[2] Lanzetta D, Cattaneo D, Pellegatta D, Cardini R. (2004). Trunk control in unstable sitting posture during functional activities in healthy subjects and patients with multiple sclerosis. Arch Phys Med Rehabil.

[3] Jeon W, Whitall J, Griffin L, Westlake KP (2021). Trunk kinematics and muscle activation patterns during stand-to-sit movement and the relationship with postural stability in aging. Gait Posture.

[4] Campbell FM, Ashburn AM, Pickering RM, Burnett M (2001). Head and pelvic movements during a dynamic reaching task in sitting: implications for physical therapists. Arch Phys Med Rehabil.

[5] Van Criekinge T, Truijen S, Schröder J (2019). The effectiveness of trunk training on trunk control, sitting and standing balance and mobility post-stroke: a systematic review and meta-analysis. Clin Rehabil.

[6] Saeys W, Vereeck L, Truijen S, Lafosse C, Wuyts FP, Van de Heyning P (2012). Randomized controlled trial of truncal exercises early after stroke to improve balance and mobility. Neurorehabil Neural Repair.

[7] Karthikbabu S, Chakrapani M, Ganeshan S, Rakshith KC, Nafeez S, Prem V (2012). A review on assessment and treatment of the trunk in stroke: a need or luxury. Neural Regen Res.

[8] Liao CF, Liaw LJ, Wang RY, Su FC, Hsu AT (2015). Electromyography of symmetrical trunk movements and trunk position sense in chronic stroke patients. J Phys Ther Sci.

[9] Dean CM, Channon EF, Hall JM (2007). Sitting training early after stroke improves sitting ability and quality and carries over to standing up but not to walking: a randomised controlled trial. Aust J Physiother.

[10] Ibrahimi N, Tufel S, Singh H, Maurya M (2010). Effect of sitting balance training under varied sensory input on balance and quality of life in stroke patients. Indian J Physiother Occup Ther Int J.

[11] Veerbeek JM, Wegen E van, Peppen R van (2014). What is the evidence for physical therapy poststroke? A systematic review and meta-analysis. PLOS ONE.

[12] Shumway-Cook A, Woollacott MH (2017).

[13] Verheyden G, van Duijnhoven HJR, Burnett M, Littlewood J, Kunkel D, Ashburn AM (2011). Kinematic analysis of head, trunk, and pelvis movement when people early after stroke reach sideways. Neurorehabil Neural Repair.

[14] Wiskerke E, van Dijk M, Thuwis R (2021). Maximum weight-shifts in sitting in non-ambulatory people with stroke are related to trunk control and balance: a cross-sectional study. Gait Posture.

[15] Van Criekinge T, Saeys W, Vereeck L, De Hertogh W, Truijen S (2018). Are unstable support surfaces superior to stable support surfaces during trunk rehabilitation after stroke? A systematic review. Disabil Rehabil.

[16] Karthikbabu S, Nayak A, Vijayakumar K (2011). Comparison of physio ball and plinth trunk exercises regimens on trunk control and functional balance in patients with acute stroke: a pilot randomized controlled trial. Clin Rehabil.

[17] Bauer CM, Nast I, Scheermesser M (2021). A novel assistive therapy chair to improve trunk control during neurorehabilitation: perceptions of physical therapists and patients. Appl Ergo.

[18] Bohannon RW (2022). Measurement of trunk muscle strength after stroke: an integrative review. Top Stroke Rehabil.

[19] Massie CL, Malcolm MP, Greene DP, Browning RC (2012). Kinematic motion analysis and muscle activation patterns of continuous reaching in survivors of stroke. J Motor Behav.

[20] Oomen NMCW, Reeves NP, Priess MC, van Dieën JH (2015). Trunk muscle coactivation is tuned to changes in task dynamics to improve responsiveness in a seated balance task. J Electromyogr Kinesiol.

[21] Thijs L, Voets E, Wiskerke E (2021). Technology-supported sitting balance therapy versus usual care in the chronic stage after stroke: a pilot randomized controlled trial. J Neuroeng Rehabil.

[22] Bauer CM, Rast FM, Bock C, Kuster RP, Baumgartner D (2018). Determination of a sagittal plane axis of rotation for a dynamic office chair. Appl Ergon.

[23] Kuster RP, Bauer CM, Oetiker S, Kool J (2016). Physiological motion axis for the seat of a dynamic office chair. Hum Factors.

[24] Kuster RP, Bauer CM, Gossweiler L, Baumgartner D (2018). Active sitting with backrest support: is it feasible?. Ergonomics.

[25] IEC. IEC 80601-2-78:2019. Available from: https://www.iso.org/cms/render/live/en/sites/isoorg/contents/data/standard/06/84/68474.html. Accessed November 9, 2020.

[26] ISO. ISO 24496:2017. Available from: https://www.iso.org/cms/render/live/en/sites/isoorg/contents/data/standard/06/68/66835.html. Accessed November 9, 2020.

[27] ISO. ISO - ISO 13485 — Medical devices. Available from: https://www.iso.org/iso-13485-medical-devices.html. Accessed November 9, 2020.

[28] Hermens HJ, Freriks B, Merletti R (1999). Roessingh Research and Development b.v.

[29] Ng JK-F, Kippers V, Richardson CA (1998). Muscle fibre orientation of abdominal muscles and suggested surface EMG electrode positions. Electromyogr Clin Neurophysiol.

[30] Bates D, Mächler M, Bolker B, Walker S (2015). Fitting linear mixed-effects models using lme4. J Stat Software.

[31] Kuznetsova A, Brockhoff PB, Christensen RHB (2017). lmerTest package: tests in linear mixed effects models. J Stat Software.

[32] CRAN R project. Procedures for personality and psychological research. Available at: https://CRAN.R-project.org/package=psych. Accessed November 30, 2021.

[33] CRAN R project. emmeans: estimated marginal means, aka least-squares means. Available at: https://CRAN.R-project.org/package=emmeans. Accessed November 30, 2021.

[34] gglpot2. ggplot2: elegant graphics for data analysis. Available from: https://ggplot2.tidyverse.org. Accessed November 30, 2021.

[35] Kiefer A, Shirazi-Adl A, Parnianpour M (1998). Synergy of the human spine in neutral postures. Eur Spine J.

[36] O'Sullivan P, Dankaerts W, Burnett A (2006). Lumbopelvic kinematics and trunk muscle activity during sitting on stable and unstable surfaces. J Orthop Sports Phys Ther.

[37] Vera-Garcia FJ, Barbado D, Flores-Parodi B, Alonso-Roque J, Elvira JLL (2013). Trunk muscle activation in spine stabilization exercises. Rev Int Med Cienc Act Fis Dep.

[38] Preuss RA, Grenier SG, McGill SM (2005). Postural control of the lumbar spine in unstable sitting. Arch Phys Med Rehabil.

[39] Yoo W (2013). Effects of an unstable dual foot support on the trunk flexion angle and RF, L4-ES, EO muscle activities during computer work. J Phys Ther Sci.

[40] De Luca A, Squeri V, Barone LM (2020). Dynamic stability and trunk control improvements following robotic balance and core stability training in chronic stroke survivors: a pilot study. Front Neurol.

[41] Olczak A (2021). Importance of core stability for coordinated movement of the human body in stroke rehabilitation. Neurol Res.

[42] Messier S, Bourbonnais D, Desrosiers J, Roy Y (2004). Dynamic analysis of trunk flexion after stroke. Arch Phys Med Rehabil.

[43] Mochizuki G, Ivanova TD, Garland SJ (2004). Postural muscle activity during bilateral and unilateral arm movements at different speeds. Exp Brain Res.

[44] Veerbeek JM, Pohl J, Luft AR, Held JPO (2022). External validation and extension of the Early Prediction of Functional Outcome after Stroke (EPOS) prediction model for upper limb outcome 3 months after stroke. PLOS ONE.

